# Transcriptional Changes in the Hookworm, *Ancylostoma caninum*, during the Transition from a Free-Living to a Parasitic Larva

**DOI:** 10.1371/journal.pntd.0000130

**Published:** 2008-01-09

**Authors:** Bennett J. D. Datu, Robin B. Gasser, Shivashankar H. Nagaraj, Eng K. Ong, Peter O'Donoghue, Russell McInnes, Shoba Ranganathan, Alex Loukas

**Affiliations:** 1 Helminth Biology Laboratory, Division of Infectious Diseases and Immunology, Queensland Institute of Medical Research, Herston, Queensland, Australia; 2 School of Molecular and Microbial Sciences, The University of Queensland, Brisbane, Australia; 3 Department of Veterinary Science, The University of Melbourne, Werribee, Victoria, Australia; 4 Department of Chemistry and Biomolecular Sciences & Biotechnology Research Institute, Macquarie University, Sydney, New South Wales, Australia; 5 Department of Science, Museum Victoria, Melbourne, Victoria, Australia; 6 Agilent Technologies, Forest Hill, Victoria, Australia; 7 Department of Chemistry and Biomolecular Sciences & Biotechnology Research Institute, Macquarie University, Sydney, New South Wales, Australia; University of Technology, Sydney, Australia

## Abstract

**Background:**

Third-stage larvae (L3) of the canine hookworm, *Ancylostoma caninum*, undergo arrested development preceding transmission to a host. Many of the mRNAs up-regulated at this stage are likely to encode proteins that facilitate the transition from a free-living to a parasitic larva. The initial phase of mammalian host invasion by *A. caninum* L3 (herein termed “activation”) can be mimicked *in vitro* by culturing L3 in serum-containing medium.

**Methodology/Principal Findings:**

The mRNAs differentially transcribed between activated and non-activated L3 were identified by suppression subtractive hybridisation (SSH). The analysis of these mRNAs on a custom oligonucleotide microarray printed with the SSH expressed sequence tags (ESTs) and publicly available *A*. *caninum* ESTs (non-subtracted) yielded 602 differentially expressed mRNAs, of which the most highly represented sequences encoded members of the pathogenesis-related protein (PRP) superfamily and proteases. Comparison of these *A. caninum* mRNAs with those of *Caenorhabditis elegans* larvae exiting from developmental (dauer) arrest demonstrated unexpectedly large differences in gene ontology profiles. *C*. *elegans* dauer exiting L3 up-regulated expression of mostly intracellular molecules involved in growth and development. Such mRNAs are virtually absent from activated hookworm larvae, and instead are over-represented by mRNAs encoding extracellular proteins with putative roles in host-parasite interactions.

**Conclusions/Significance:**

Although this should not invalidate *C*. *elegans* dauer exit as a model for hookworm activation, it highlights the limitations of this free-living nematode as a model organism for the transition of nematode larvae from a free-living to a parasitic state.

## Author Summary

Hookworms are soil-transmitted nematodes that parasitize hundreds of millions of people in developing countries. Here we describe the genes expressed when hookworm larvae make the transition from a developmentally arrested free-living form to a tissue-penetrating parasitic stage. *Ancylostoma caninum* can be “tricked” into thinking it has penetrated host skin by incubating free-living larvae in host serum – this is called “activation”. To comprehensively identify genes involved in activation, we used suppressive subtractive hybridization to clone genes that were up- or down-regulated in activated larvae, with a particular focus on up-regulated genes. The subtracted genes, as well as randomly sequenced (non-subtracted) genes from public databases were then printed on a microarray to further explore differential expression. We compared predicted gene functions between activated hookworms and the free-living nematode, *Caenorhabditis elegans,* exiting developmental arrest (dauer), and found enormous differences in the types of genes expressed. Genes encoding secreted proteins involved in parasitism were over-represented in activated hookworms whereas genes involved in growth and development dominated in *C. elegans* exiting dauer. Our data implies that *C. elegans* dauer exit is not a reliable model for exit from developmental arrest of hookworm larvae. Many of these genes likely play critical roles in host-parasite interactions, and are therefore worthy of pursuit for vaccine and drug development.

## Introduction

Parasitic nematodes are of considerable medical, veterinary and agricultural importance. For example, it is estimated that the morbidity attributable to hookworms, *Trichuris* and *Ascaris*, the three most prevalent parasitic nematodes in humans globally, could be as high as 39 disability adjusted life years (DALY) [1]. This assessment takes into account the long-term impact of infection on cognitive and physical development and the overall health of the host. World-wide, ∼1.3 billion people are infected with at least one of these geohelminths [2]. The prevalence of the human hookworms, *Ancylostoma duodenale* and *Necator americanus*, alone approaches 740 million, with the foci predominantly within Asia, sub-Saharan Africa, and Latin America [3].

Facultative developmental arrest in the free-living nematode, *Caenorhabditis elegans*, can occur transiently in the first larval stage (L1) as well as for prolonged periods at the L3 stage. Developmental arrest (often referred to as the dauer stage) in the L3 is triggered in response to conditions, such as crowding, scarcity of food and elevated temperature [4]. When the environment improves, worms exit the arrest to resume development. However, under permissive conditions, arrest is bypassed and adult and reproductive development is favoured. For many parasitic nematodes, arrest at the L3 facilitates survival in the environment. The exit from arrest marks the return to growth and development as well as the transmission of the parasite to its host. Larvae invade a suitable host and undergo a migration through particular tissues to then establish in a target organ and complete the life cycle or arrest in specific tissues. The infective L3 of many parasitic nematodes produce mRNAs which are thought to relate to invasion, migration, and/or survival [5]–[10]. Therefore, the characterization of mRNAs transcribed in the L3 during its transition from the free-living to the parasitic stage may aid in the identification of genes associated with these processes. An attractive parasite model in which to experimentally study this transition is the dog hookworm, *Ancylostoma caninum*, for which an *in vitro* serum-stimulation assay exists [11].

Several molecular aspects associated with serum stimulation have been investigated previously in *A. caninum*. Some researchers have focused on the release of activation-associated proteins; these molecules include the pathogenesis related protein (PRP) superfamily members *Ac*-ASP-1 [12] and *Ac*-ASP-2 [13], and the metalloprotease *Ac*-MTP-1 [14], all of which represent the most abundant excreted/secreted proteins released by serum-stimulated (activated) L3. Other workers have studied activation-associated genes of hookworms using a transcriptomic approach. For instance, Mitreva et al. [8] generated expressed sequence tags (ESTs) for *A*. *caninum* (serum stimulated, unstimulated and tissue-arrested L3) and *A*. *ceylanicum* (unstimulated L3 and adults), being the first systematic study of genes associated with the host invasion process. However, this study had some limitations in that (1) comparative analyses made between larval stages were qualitative rather than quantitative; (2) some of the observed differences in the abundance of ESTs between activated and non-activated *A*. *caninum* L3 seemed to be attributable to differences in the procedures employed for the construction of the cDNA libraries from these life-cycle stages; and (3) the study included a relatively small number of randomly generated sequences available at the time for *A*. *caninum* (n = 3840) and *A*. *ceylanicum* (n = 3149). Moser et al. [7] addressed the first two points by conducting a quantitative microarray analysis of *A. caninum* genes associated with the transition to parasitism, focusing on decreased transcription after serum stimulation (i.e., those mRNAs which are “switched off” or reduced in transcription upon host entry). However, this study was also limited to known ESTs available in the public databases.

To infer the mRNAs involved in the infective process of *A*. *caninum*, we conducted herein a quantitative study of all known *A*. *caninum* sequences as well as newly identified genes discovered through suppressive-subtractive hybridisation (SSH) of activated *versus* non-activated L3 of *A. caninum*. The method of SSH was employed to selectively enrich differentially transcribed genes [15]. In summary, 242 potentially up-regulated and 109 potentially down-regulated mRNAs were identified by SSH. There were many mRNAs that were differentially expressed but not identified by SSH, although this might be a function of the number of clones randomly sequenced from our subtracted libraries. The final repertoire of activation-associated genes consisted of 240 up-regulated and 362 down-regulated mRNAs. Among these nearly 600 activation-associated genes were numerous (often substantially up-regulated) mRNAs encoding PRPs and three of the major catalytic classes of proteases (metallo-, cysteine, and aspartic). Several mRNAs encoding novel secreted proteins without any known homologues were also identified. These mRNAs, if demonstrated to be integral to the parasitic process, could represent a new generation of potential vaccine antigens and drug targets against hookworms.

## Methods

### Parasitological methods

**Figure 1:**
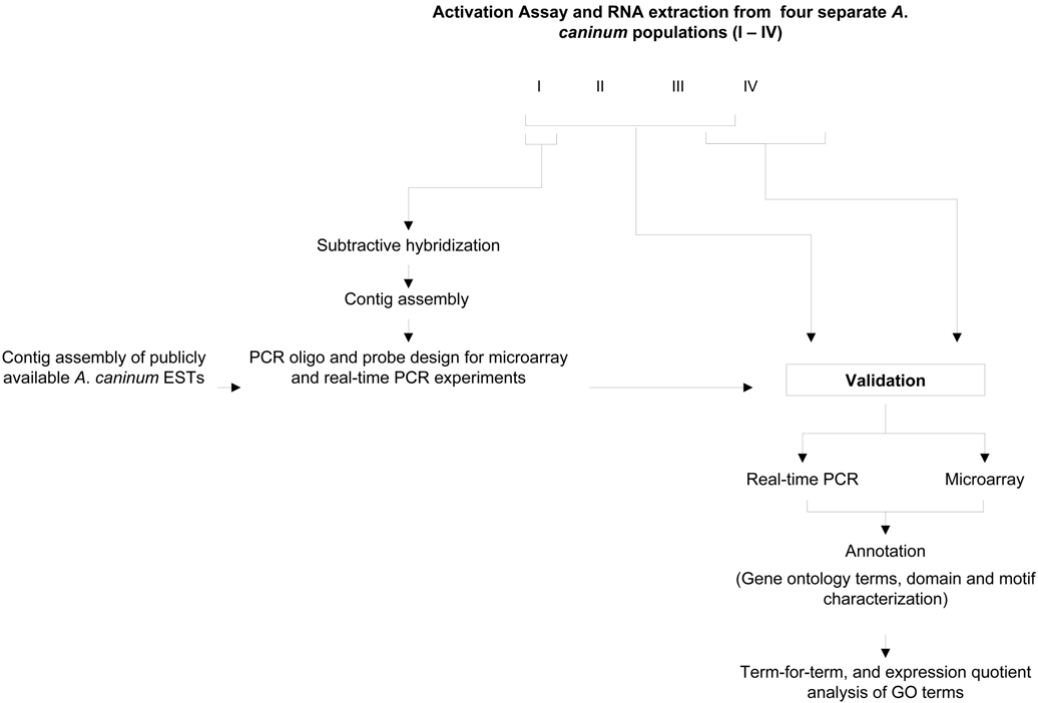
Experimental design. Suppressive subtractive hybridisation (SSH) was performed on one group of *Ancylostoma caninum* larvae and subsequently validated by real-time PCR and microarray experiments using three additional groups of larvae from separate infections. Bioinformatic analysis was conducted on the validated pool of activation-associated genes.

*A*. *caninum* L3 were isolated from the faeces of stray dogs in the greater Brisbane area and surrounding towns in Queensland, Australia, using a standard charcoal coproculture method. Cultures were incubated at ∼23°C in a humidified chamber for one week, after which L3 were concentrated using a modified Baermann technique and purified through a nylon filter (20 µm). Larvae were stored for up to four weeks in 50 mM Na_2_HPO_4_, 22 mM KH_2_ PO_4_, 70 mM NaCl, pH 6.8 [16] in 12.5 cm^2^ vented tissue culture flasks in the dark at room temperature until use. In total, four separate groups of L3 representing four separate infections from different geographical locations were obtained. The first group was used for SSH and time course studies, whereas the others were employed as biological replicates in microarray validation and real-time PCR analyses (Figure 1).

### Verification of the specific identity of *A. caninum* L3

The specific identity of the parasite material was confirmed by PCR amplification of the first and second internal transcribed spacers (ITS-1 and ITS-2) of nuclear ribosomal DNA (as described by [17]) and automated sequencing (using BigDye chemistry, ABI). The sequences determined were required to be identical to those with GenBank accession numbers Y19181 (ITS-1) and AJ001591 (ITS-2).

### *In vitro* activation of larvae

Prior to *in vitro* activation (serum-stimulation), ensheathed L3 were incubated in 1% HCl for 30 min at ∼23°C and then resuspended in RPMI-C (RPMI-1640 tissue culture medium supplemented with 25 mM HEPES (pH 7.0), 100 IU/ml of penicillin, 100 µg/ml of streptomycin, and 40 µg/ml of gentamycin) [12]. To each well of a 24-well tissue culture plate, 5,000 L3 were added. For the SSH, a total of 40,000 L3 were activated in 15% serum and 25 mM S-methylglutathione in RPMI-C, whereas 25,000 L3 were incubated in RPMI-C alone (non-activated control). Five thousand L3 were sampled at each of four time points during the activation (1, 6, 13, and 24 h), in order to perform a time-course analysis of transcripts using real-time PCR (described in “Validation of transcription *via* real-time PCR”). The same number of non-activated control L3 were separately prepared for this analysis, leaving 20,000 activated and 20,000 non-activated worms for SSH.

For the microarray analysis, activated and non-activated L3 (50,000 of each) were prepared from each of two separate populations of *A. caninum*. Also, activated and non-activated L3 (5,000 of each) were prepared for real-time PCR. For the *in vitro* activation, L3 were incubated overnight at 37°C in 5% CO_2_; pharyngeal pumping in activated L3 was verified by feeding ∼100 of them with FITC-BSA (10 mg/ml) for 3 h and fluorescence was detected using a Leica DM IRB inverted microscope with a Leica DC 500 high-resolution digital camera [11]. Activated and non-activated L3 were each washed twice in phosphate-buffered saline (PBS, pH 7.4; 23°C) and immediately frozen at −80°C. For RNA isolation, larvae were resuspended in 100 µl of Trizol reagent and homogenized in a 1.5 ml tube using an RNase-free, disposable, in-tube pestle and subjected to three rapid (1 min) freeze/thaw cycles. Trizol was added to a final volume of 500 µl, before snap freezing in liquid nitrogen. These samples were stored for ≤1 month at −80°C before RNA was isolated.

### RNA isolation

Frozen samples of L3 in Trizol were brought to 4°C and centrifuged (16,000×*g* at the same temperature) for 10 min to remove insoluble debris and residual genomic DNA. RNA was then extracted with chloroform, precipitated with isopropanol, washed with absolute ethanol and resuspended in 50 µl of RNAse-free water. Each RNA sample was treated with 2 U of *DNase* I (Promega) prior to heat denaturation of the enzyme (75°C for 5 min) and frozen immediately at −80°C. The integrity of RNA was verified to have an RNA Integrity Number >8.0 using an Agilent 2100 Bioanalyzer and RNA 6000 LabChip Kit (Agilent Technologies). RNA used for microarray analysis was stored as an ethanol precipitate in 75% ethanol at −80°C.

### Suppression subtractive hybridisation (SSH)

First strand cDNA was synthesized from 1 µg of total RNA using the SuperSmart cDNA synthesis kit (Clontech), according to the manufacturer's protocol. Subsequently, double stranded cDNA was produced through 17 rounds of PCR amplification and purified by phenol:chloroform:isoamyl alcohol (25:24:1) extraction, followed by sodium acetate precipitation. SSH was carried out using the PCR Select cDNA subtraction kit (Clontech) according to the manufacturer's protocol. Briefly, cDNA from activated or non-activated *A. caninum* L3 was digested with the endonuclease *Rsa* I and ligated to adapters, yielding tester cDNAs for each treatment. Activated tester cDNA was denatured and allowed to re-hybridize in an excess of non-activated “driver” cDNA. This hybridization was termed the forward subtraction and enriched for cDNAs with a higher abundance in activated worms. A reverse subtraction was also performed which enriched for cDNAs which were more abundant in the non-activated worms. Also, an unsubtracted control was prepared according to the standard protocol. Hybridized cDNAs were amplified *via* two rounds of PCR (according to the recommended protocol), purified by spin-column (QIAGEN) and then cloned into the plasmid vector pGEM-T (Promega). Chemically competent *Escherichia coli* (TOP 10) were transformed and grown for 8 h at 37°C in Luria Bertani medium (LB) with 100 µg/ml ampicillin. Stocks were stored in glycerol (20%) at –80°C. Immediately prior to sequencing, 50 µl of LB (22°C) was added to each *E. coli* stock and grown overnight on LB agar plates containing 100 µg/ml of ampicillin. Recombinant colonies were isolated by blue/white selection and then arrayed on a grided LB plate containing 100 µg/ml of ampicillin. Inserts amplified using the TempliPhi DNA Sequencing Template Amplification kit (GE Healthcare Life Sciences) were sequenced unidirectionally using the T7 vector primer in an ABI 3730X1 DNA analyser.

### EST sequencing and bioinformatic analysis

The chromatograms for all raw ESTs were inspected and processed to remove poor quality sequence, with subsequent removal of contaminating vector sequences using BioEdit software v.7.0.1. Following this pre-processing, ESTs were organized into contigs and clusters through an iterative approach using the Cap contig assembly facility in BioEdit under strict conditions, requiring at least a 100 bp overlap and 95% identity among sequences. The resultant contigs and singletons were named according to a simple convention. A “C” in the sequence name identifies sequences composed of multiple ESTs while singletons are indicated with an “S”. Sequences from the forward-subtracted library have four digit identifiers, whereas those from the reverse-subtracted library have three digits. For example, *Ac*_SSH_C_0056 indicates that the SSH sequence 0056 is composed of multiple ESTs from the forward subtracted library. SSH sequences were compared with existing sequences in GenBank and Wormbase (www.wormbase.org) *via* BLASTx through NCBI (www.ncbi.nlm.nih.gov/BLAST/) and WU-BLAST (www.ebi.ac.uk/blast2). Alignments were considered statistically significant if an E- or P-value was ≤1×10^−5^. Neural networks and hidden Markov models were used to predict signal peptides and transmembrane domains by way of the SignalP 3.0 (www.cbs.dtu.dk/services/SignalP/) and TMPred (www.ch.embnet.org/software/TMPRED_form.html) interfaces, respectively. Conserved protein motifs of activation-associated ORFs were identified using the InterProScan website (www.ebi.ac.uk/InterProScan). Potential proteases were classified using the MEROPS protease database (http://merops.sanger.ac.uk/index.htm). Contigs were also mapped to gene ontology (GO) terms based on sequence similarity using the BLAST2GO platform (www.blast2go.de) which compares all contigs with sequences available in several databases, including Wormbase and Uniprot [18]. Only BLASTx hits with a maximum E-value ≤1×10^−10^ and a minimum of 50% similarity (default software settings) were selected for annotation. A modified one-tailed Fisher exact test based on a hypergeometric distribution was employed in the identification of GO terms for differentially transcribed genes, which were significantly over-represented [19]. This assessment was made relative to the total number of *A. caninum* genes which had been GO-annotated. Setting the “false discovery” rate limit to 0.5 aided in controlling for multiple testing errors [18].

### Microarray: probe design

Sequence data for 9,618 *A*. *caninum* ESTs were obtained from the Washington University Genomics Department *via* the NCBI sequence database (http://www.ncbi.nlm.nih.gov/Genbank/index.html). Chromatograms were pre-processed with the Phred software [20],[21] and organized into contigs and clusters with the Cap3 contig assembly program [22], employing a minimum sequence overlap length of 30 bases and an identity threshold of 95%. Contigs (n = 1311) were assembled from the ESTs and are hereafter designated with “Contig”, followed by a number between 1 and 1311. The remaining singletons were filtered by BLAST E-values (<0.001) to remove potentially spurious sequences and are henceforth referred to by their GenBank accession number. In total, 2,889 individual sequences were identified from the total EST dataset for *A*. *caninum*. Sequences representing individual clusters assembled from the sequence data from the forward and reverse subtracted cDNAs as well as the publicly available repository were combined. The combined dataset (a total of 3,100 representative sequences) were submitted for the design of 60-mer oligonucleotides using eArray (Agilent). A total of 9,288 oligonucleotides (3 per target) were proposed for 3,096 contigs. Of these oligonucleotides, 3,443 possessed a non-self perfect match, resulting in 5,845 representing 1,967 genes suitable for microarray analysis. These 5,845 oligonucleotide probe sequences were electronically submitted using eArray for ink-jet *in-situ* synthesis onto glass slides by Agilent Technologies.

### Microarray: cRNA preparation, hybridization and data analysis

To generate cRNA, 200 ng of total RNA extracted from each activated and non-activated L3 population of *A. caninum* was reverse transcribed and simultaneously labelled with Cy3 or Cy5 (Agilent). Immediately prior to hybridisation, 500 ng of labelled cRNAs from each of activated and non-activated worms were quantified using a NanoDrop ND-1000 UV-VIS spectrophotometer (NanoDrop), assessed for size distribution and Cy5-dye incorporation using an Agilent 2100 Bioanalyzer and RNA 6000 LabChip Kit (Agilent), mixed together and fragmented. The cRNA from the combined treatments for each population was hybridised to the array in duplicate, with the second hybridisation representing a dye swap to control for any bias in signal intensity between the two dyes. Hybridisations and washes were conducted as per Agilent's Two-colour Gene Expression Hybridisation protocol version 5.0.1. Briefly, 250 µl of hybridisation solution was applied and the microarrays were hybridised for 17 hours at 65°C, 10 rpm. Slides were then washed for 1 minute in Wash Buffer 1 (RT), 1 minute in Wash Buffer 2 (37°C), 1 minute in Acetonitrile (RT) and 30s in Stabilisation and Drying Solution (RT). Slides were scanned using a DNA Microarray Scanner (Agilent). Scanning and feature extraction were performed using Feature Extraction software version 9.1 (extraction protocol GE2-v4_91; Agilent). During extraction, signal intensities were Linear and Lowess-normalized, dye-corrected, and adjusted for local background. Data handling and analysis were carried out using the program SAS v.8.0 (SAS Institute). Processed signal intensities for each probe were averaged across genes, replicates and populations for comparison between treatments by a two-sided *t-*test with a Type I error rate of 0.01. Only signals differing by at least 1.5 fold (P≤0.01) for each population were considered to represent molecules differentially transcribed in *A. caninum* as a consequence of serum stimulation *in vitro*. The effects of dye and probe on the mean signal were assessed graphically. Fold changes in hybridisation were expressed as log_2_-transformed ratios. The absolute log_2_ ratios within each level-three GO category were averaged and divided by the mean absolute log_2_ ratio of all spots on the chip to derive an expression quotient (EQ). The EQ provides an indication of the degree of differential expression associated with a specific GO term.

### Validation of transcription *via* real-time PCR

Reverse transcription real-time PCR was used for the validation of microarray data and for studying levels of transcription in L3 at different time points during the course of serum stimulation *in vitro*. Ten target sequences were chosen at random and seven others were selected to represent contigs with high, medium and low levels of hybridisation in the microarray. The sequences of all of the primers used in the real-time PCR are listed in Table S2. The single-stranded cDNA template was quantified spectrophotometrically and diluted to an appropriate concentration (2 ng/µl). Two ng of cDNA from each activated and non-activated *A. caninum* L3 population were subjected to PCR in the presence of 100 nM of the forward and reverse primers in 1× Platinum SYBR Green qPCR SuperMix-UDG (Invitrogen). All experiments were repeated three times with two replicates in each using a Rotor-Gene 6000 Series 2-Plex real-time PCR thermal cycler (Corbett Life Science) employing the following cycling parameters: 50°C for 2 min, 95°C for 2 min, and 40 cycles of 95°C for 15 sec and 60°C for 30 sec. A melt curve analysis was performed from 60°C to 95°C in 1°C intervals to demonstrate the specificity of each amplicon and to identify the formation of primer dimers. Amplicons were also inspected on a 1.2% agarose gel and subjected to automated sequencing to prove their identity. Fold changes in transcripts between activated and non-activated L3 were normalized to the 60S acidic ribosomal protein gene (accession number BF250585) [23] according to an established method [24],[25]. The standard error of the log_2_ ratios was calculated from the error of the crossing points and observed reaction efficiencies propagated through the calculation of the ratio. Non-parametric statistical inference testing of log_2_-transformed ratios was performed using a pairwise fixed reallocation randomisation approach with 10,000 simulations which calculated the probability of observing ratios of randomly assigned control and treatment pairs greater than or equal to the treatment effect observed.

## Results

### Activation and feeding

**Figure 2:**
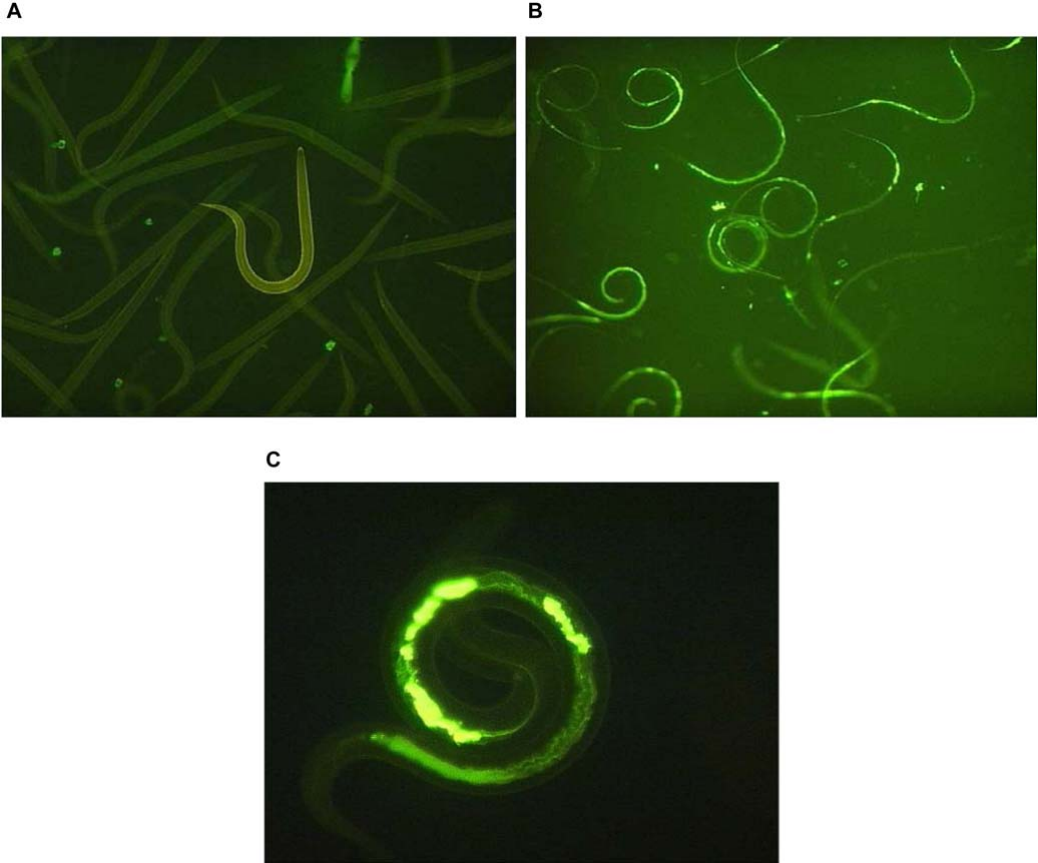
Example of *A. caninum* third-stage larvae (L3) ingesting fluorescein-conjugated bovine serum albumin (FITC-BSA) following serum-stimulation activation *in vitro*. Ensheathed (non-activated) L3 do not ingest FITC-BSA (A) whereas serum-stimulation activated L3 ingest the dye and exhibit strong fluorescence from the gut (B-100×magnification; C-400×magnification).

Approximately 120,000 activated and 120,000 non-activated L3 of *A. caninum* were prepared for RNA extraction. As evidenced by the ingestion of FITC-BSA, >95% of all activated L3 resumed feeding, whereas <4% of the non-activated L3 fed (Figure 2). L3 that failed to feed in the presence of the serum stimulus or those that fed in the absence of the stimulus could not be separated from each other.

### SSH cDNA libraries and general characteristics of the ESTs

The RNAs from activated and non-activated L3 were extracted, processed, subjected to forward and reverse SSH and cloned into a plasmid vector. A total of 958 sequencing reactions from the forward library and 171 from the reverse library yielded high quality ESTs. The sequences were deposited in GenBank (accession numbers ES671894–ES672870) and dbEST (accession numbers 46880363 – 46881339) databases. Clustering of these SSH ESTs yielded totals of 242 forward subtracted and 109 reverse-subtracted contigs (Table 1). Approximately half of all forward subtracted sequences were represented by a single EST, although this figure was ∼80% for reverse subtracted sequences. The minimum and maximum lengths of the ESTs were 100 bp and 1500 bp, respectively, with the forward subtracted contigs being slightly larger (571 bp) than those from the reverse subtracted contigs (499 bp). Contigs assembled from the publicly available ESTs for *A*. *caninum* had a similar size distribution compared with those assembled from the subtracted ESTs.

**Table 1:** Cluster summary of Suppression Subtractive Hybridization expressed sequence tags

Source	Number of sequences				Sequence length		
	ESTs	Contigs	Singletons	Total	Mean	Min	Max
Forward subtracted library	958	110	132	242	570.6	96	1454
Reverse subtracted library	171	18	91	109	499.4	100	1492
Population	9618	1311	1578	2889	495.8	108	1984

The term “population” refers to the 9618 publicly available *A. caninum* expressed sequence tags.

From the SSH-derived ESTs, almost 30% of all contigs from both libraries lacked significant sequence similarity to any of the ESTs generated previously for this species [8]. Furthermore, 64–70% of the ESTs from the forward and reverse subtracted libraries respectively did not exhibit significant similarity (at both the nucleotide and protein levels) to sequences within the databases queried (GenBank, EMBL and WormBase) (Table 2). Most mRNAs identified by SSH (63.8%) had a predicted ORF of >50 amino acids. Of these, 19% from the forward subtracted contigs had ORFs with a predicted signal sequence as compared with 6% from the reverse subtracted contigs.

**Table 2:** Characteristics of Suppression Subtractive Hybridization sequences

	Total		Forward SSH		Reverse SSH	
	n	%	n	%	n	%
New *A*. *caninum* sequences	103	29.3	69	28.5	34	31.2
New and unique sequences	68	19.4	44	18.2	24	22.0
Similar sequences in GenBank (nr)	213	60.7	147	60.7	66	60.6
Similar nematode ESTs in dbEST	193	55.0	136	56.2	57	52.3
ORF ≥50 amino acids	224	63.8	174	71.9	50	45.9
Predicted signal peptide	53	15.1	46	19.0	7	6.4
Predicted transmembrane domain	18	5.1	13	5.4	5	4.6
Gene ontology annotation	110	31.3	76	31.4	34	31.2

*A*. *caninum* sequences identified by Suppression Subtractive Hybridization (SSH) were considered to be new if they shared less than 95% identity and at least 30 base pairs of overlap with any of the publicly available *A*. *caninum* expressed sequence tags. SSH sequences were considered to be unique to *A*. *caninum* if a similar sequence could not be identified from another species (E-value cut-off, 1×10^−6^) in dbEST, GenBank, or Swissprot.

### Microarray validation of SSH

**Figure 3:**
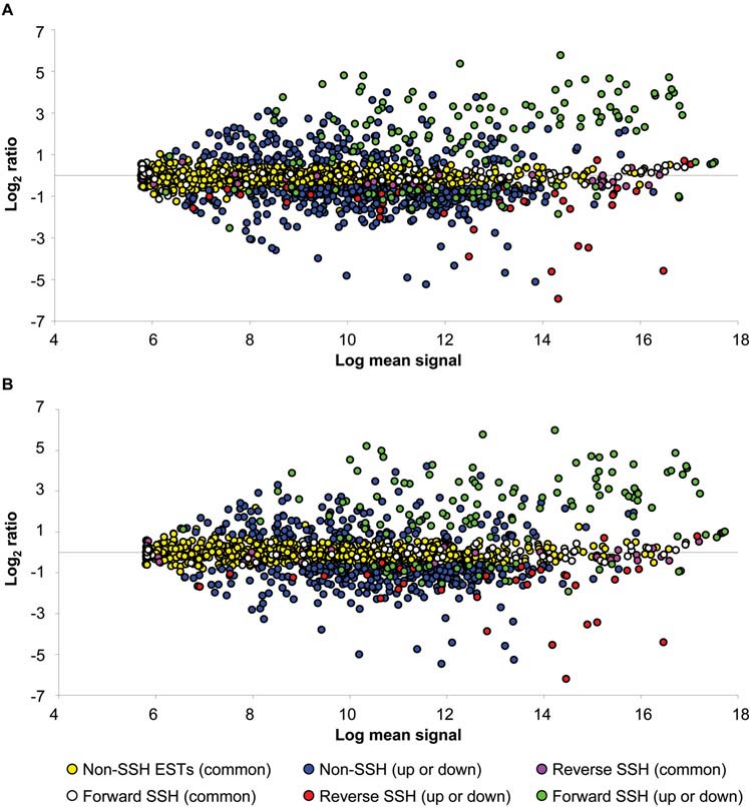
Magnitude (M) *versus* Amplitude (A) plot of array data summarized by population. Data points are colour-coded to highlight the difference between significant and non-significant log ratios as well as agreement with predictions based on results achieved using suppressive subtractive hybridisation. Yellow, pink, and white points represent sequences with log_2_ ratios which were not significantly different from zero (P>0.01). All other points denote sequences with log_2_ ratios significantly different from zero (P<0.01). Results for worms from Group III (A) and Group IV (B).

The differential hybridisation of forward and reverse subtracted contigs identified by SSH was verified using a custom designed oligonucleotide microarray. In order to assess the sensitivity and specificity of SSH, we clustered the entire *A*. *caninum* EST dataset (9,618 ESTs) and submitted the union of the public ESTs and the SSH contigs for oligonucleotide design. Slides were hybridised with cRNA derived from two separate populations of hookworms (Groups III and IV; Figure 1). The number of L3 obtained from Group III was sufficient to produce two separate pools, serving as a technical replicate for RNA extraction. In total, eight separate hybridisations were performed, one for each of the three RNA samples, plus a dye swap as well as two self-hybridisations, in which Cy3 and Cy5 probes generated from the same RNA stock were used together to hybridise to the slide. For the two populations of *A*. *caninum* L3 used, the response to serum stimulation was very similar, as can be seen from the Magnitude (M) *versus* Amplitude (A) plots generated for each population (Figure 3). The three different oligonucleotides designed for each target yielded consistent log_2_ ratios among the 50 *A*. *caninum* control genes (data not shown). Similar log_2_ ratios were also observed between arrays and dye-swaps (Figure S1). Furthermore, real-time PCR analysis, performed on 17 randomly chosen SSH-derived sequences, demonstrated the validity of the microarray data. However, the microarray consistently under-estimated the log_2_ ratio for highly abundant mRNAs, most likely attributable to probe saturation at both the 100% and 50% scans (data not shown). The data discussed in this publication have been deposited in NCBIs Gene Expression Omnibus (http://www.ncbi.nlm.nih.gov/geo/) and are accessible through GEO Series accession number GSE8155

### Activation associated mRNAs

In total, 602 mRNAs were associated with log_2_ transcription ratios significantly greater than zero (P≤0.01). A total of 103 mRNAs new to *A. caninum* (not in the public databases prior to this study) were identified by SSH, of which 79 had microarray data available. More than 60% of these mRNAs exhibited no significant similarity to any sequences other than *A*. *caninum* nor did they have any homologues/orthologues in the publicly available sequences for the congeneric hookworm, *A*. *ceylanicum*, or other strongylid nematodes. Sixty-three percent of unique genes, which were up-regulated upon serum stimulation, possessed a signal sequence, in comparison with 33% for down-regulated mRNAs (Table 3). The ten most abundant mRNAs in activated and non-activated L3 are listed in Table 4. Cytochrome *c* oxidase large subunit of nuclear ribosomal RNA and two novel mRNAs were amongst the most highly expressed mRNAs in both activated and non-activated larvae, with all but the two novel mRNAs being slightly, albeit significantly (P≤0.01) up-regulated upon stimulation. One of the most abundant mRNAs in non-activated larvae was *Ac-mtp-1*, encoding a metalloprotease involved in skin penetration [26], and this was the only molecule to also exhibit dramatic differential transcription upon stimulation (Table 4).

**Table 3:** Characterization of newly identified *A*. *caninum* sequences

	SSH Subtracted library		
	Totals	Forward	Reverse
No similar sequences beyond *A*. *caninum*	50	32	18
ORF≥50	17	14	3
Predicted signal peptide	6	6	0
Predicted transmembrane domain	1	1	0
Similar to existing *A*. *caninum* ESTs (dbEST)	5	3	2
Similar sequences from GenBank (nr)	0	0	0
Interpro/Swissprot annotations	2	2	0
Similar sequences from other nematodes	29	21	8
ORF≥50	23	18	5
Predicted signal peptide	2	2	0
Predicted transmembrane domain	2	1	1
Similar to other nematode ESTs (dbEST)	8	5	3
Similar sequences from GenBank (nr)	29	21	8
Interpro/Swissprot annotations	4	3	1

103 new sequences were identified by suppression subtractive hybridization, 79 of which have microarray expression data available. These sequences are divided into those that are similar to sequences from other nematodes and those that are restricted to *A*. *caninum*.

**Table 4:** Most highly expressed mRNAs in activated and non-activated L3

GeneName	Log_2_ signal	Species	Accession	E-value	Classification
***Activated AcL3***					
*Ac_*SSH_C_0047	17.9 (1.08)	*A*. *caninum*	AW700368	5×10^−91^	Cytochrome oxidase subunit III
*Ac*_SSH_C_006	17.9 (1.05)	*A*. *caninum*	AW735246	8×10^−82^	Cytochrome oxidase subunit I
*Ac_*SSH_C_0048	17.8 (0.68)	*A*. *caninum*	AW588404	8×10^−54^	Large subunit rRNA
BQ666258	17.9 (1.02)	*C*. *briggsae*	CAE68374	2×10^−20^	Hypothetical protein cbg14130
Contig1161	17.8 (1.25)				
*Ac_*SSH_C_0028	17.8 (1.08)	*A*. *caninum*	BQ25205	7×10^−84^	PRP-like protein
*Ac_*SSH_C_0009	17.7 (1.07)	*A*. *caninum*	BM130138	2×10^−81^	PRP-like protein
*Ac_*SSH_C_0019	17.7 (1.02)	*H*. *contortus*	Z69345	3×10^−103^	Cysteine proteinase
*Ac_*SSH_C_0008_A	17.6 (1.22)	*A*. *caninum*	BQ666406	3×10^−109^	PRP-like protein
SSH Contig 0140	17.6 (1.27)	*P*. *pacificus*	BI500287	7×10^−90^	PRP-like protein
***Non-activated AcL3***					
*Ac*_SSH_C_015	17.4 (0.77)	*A*. *caninum*	AW700351	1×10^−44^	*Ac*-*mtp*-*1*
*Ac*_SSH_S_0190	17.2 (0.82)	*A*. *caninum*	BQ667723	4×10^−79^	Metallothionein
*Ac*_SSH_S_0320	17.2 (0.81)	*A*. *caninum*	AW588291	2×10^−39^	Glycerol kinase
*Ac*_SSH_C_0048	17.2 (0.53)	*A*. *caninum*	AW588404	8×10^−54^	Large subunit rRNA
*Ac*_SSH_C_0047	17.2 (0.84)	*A*. *caninum*	AW700368	5×10^−91^	Cytochrome oxidase subunit III
BQ666258	17.2 (0.70)	*C*. *briggsae*	CAE68374	2×10^−20^	Hypothetical protein cbg14130
*Ac*_SSH_C_006	17.2 (0.81)	*A*. *caninum*	AW735246	8×10^−82^	Cytochrome oxidase subunit I
Contig1161	17.1 (0.82)				
*Ac*_SSH_C_0122	16.8 (0.85)	*A*. *caninum*	AW181373	8×10^−27^	Elongation factor 2
*Ac*_SSH_C_0051	16.7 (0.76)	*N*. *americanus*	BU088734	9×10^−23^	Large subunit rRNA (rpl-7A)

Log_2_ signal intensities are reported for the most highly expressed genes as evidenced by microarray analysis. Values in parentheses are standard errors. Sequence similarity searches were conducted through dbEST, NCBI, and Swiss-prot using a translated query. All accession numbers are for GenBank.

The types of proteins encoded by mRNAs that were up-regulated upon serum stimulation are very different from those that were down regulated (Tables 5 and 6). Among the 30 most highly up-regulated mRNAs, 17 encoded members of the PRP superfamily [27]. In addition >30% of these mRNAs were predicted to encode secreted proteins. In contrast, most mRNAs that were highly down regulated upon serum stimulation did not possess signal sequences and represented a more diverse group of molecules, including two heat-shock proteins, three PRPs, several novel sequences and a cytochrome P450. Thirteen mRNAs encoding proteases representing all four mechanistic classes, as determined from the MEROPS database [28], were also differentially expressed following serum stimulation (Table 7). In general, the most abundantly represented group of mRNAs associated with activation was the PRPs. Sixty-one different PRP transcripts were identified among the publicly available ESTs and the SSH dataset herein. Thirty-two of these PRPs were associated with activation, 21 of which shared greater than 50% amino acid identity with at least one of the other activation-associated PRPs. All but three of the activation-associated PRPs, *Ac*-*asp*-*2* (AW626807), an mRNA similar to *Ac*-*asp*-*1* (SSH Contig 017), and un-clustered *A*. *caninum* EST (BQ667555), were up-regulated upon serum stimulation.

**Table 5:** Most highly up-regulated mRNAs associated with serum stimulation

GeneName	Log_2_ Ratio (SE)	Species	Accession	E-value	Classification
*Ac*_SSH_C_0032	5.8 (0.54)	*A*. *caninum*	BQ667276	3×10^−84^	Novel (SP)
*Ac*_SSH_C_0042_B	5.5 (0.14)	*A*. *caninum*	BQ666426	3×10^−103^	PRP superfamily member (SP)
*Ac*_SSH_C_0027	5.0 (0.44)	*A*. *caninum*	BQ667497	8×10^−68^	PRP superfamily member
*Ac*_SSH_C_0069	4.9 (0.51)	*A*. *caninum*	BQ666908	2×10^−60^	Weak similarity to boophilin
*Ac*_SSH_C_0008_A	4.8 (0.79)	*A*. *caninum*	BQ666406	3×10^−109^	PRP superfamily member (SP)
*Ac*_SSH_C_0042_A	4.7 (0.12)	*A*. *caninum*	BQ666554	1×10^−95^	PRP superfamily member (SP)
*Ac*_SSH_C_0099	4.6 (1.11)				(SP)
*Ac*_SSH_C_0144	4.5 (0.43)	*A*. *caninum*	AF089728	2×10^−59^	PRP superfamily member
*Ac*_SSH_C_0006	4.4 (0.61)	*A*. *caninum*	CW76671	1×10^−89^	PRP superfamily member (SP)
*Ac*_SSH_C_0081	4.4 (0.50)	*A*. *ceylanicum*	AY136548	1×10^−105^	PRP superfamily member
*Ac*_SSH_C_0044	4.4 (0.59)				
*Ac*_SSH_C_0065	4.2 (0.37)	*A*. *ceylanicum*	AAR03712	3×10^−13^	PRP superfamily member (SP)
*Ac*_SSH_C_0019	4.2 (0.34)	*H*. *contortus*	Z69345	3×10^−103^	Cysteine proteinase (SP)
*Ac*_SSH_C_0041	4.1 (0.41)	*A*. *caninum*	BQ667185	1×10^−79^	Apyrase (SP)
*Ac*_SSH_C_0009	4.0 (0.37)	*A*. *caninum*	BM130138	2×10^−81^	PRP superfamily member (SP)
*Ac*_SSH_C_0118	4.0 (0.45)	*A*. *caninum*	BM130091	8×10^−27^	PRP superfamily member (SP)
*Ac*_SSH_S_0350	4.0 (0.84)	*A*. *caninum*	BQ666550	2×10^−86^	PRP superfamily member (SP)
BI744483	4.0 (0.42)	*C*. *briggsae*	CAE72985	2×10^−34^	Hyp. protein cbg20329
*Ac*_SSH_C_0131_B	4.0 (0.42)	*A*. *caninum*	BQ667320	6×10^−44^	Novel
BQ666642	4.0 (0.41)				
*Ac*_SSH_C_0040	3.9 (0.62)	*A*. *caninum*	BQ667639	2×10^−67^	PRP superfamily member (SP)
*Ac*_SSH_C_0017	3.9 (0.09)	*A*. *caninum*	BQ667639	6×10^−93^	PRP superfamily member (SP)
*Ac*_SSH_S_0131_A	3.9 (0.35)	*A*. *caninum*	BQ667320	2×10^−22^	Novel
*Ac*_SSH_C_0140	3.8 (0.40)	*P*. *pacificus*	BI500287	7×10^−90^	PRP superfamily member (SP)
*Ac*_SSH_S_0158	3.8 (0.51)	*A*. *caninum*	BQ666836	6×10^−46^	PRP superfamily member (SP)
BQ667750	3.8 (0.45)	*C*. *elegans*	CAA94349	2×10^−7^	Hyp. protein f49e11.5
*Ac*_SSH_C_0086	3.7 (0.31)				
BM077611	3.6 (0.32)				(SP)
*Ac*_SSH_C_0143	3.5 (1.07)	*A*. *ceylanicum*	AY288090	2×10^−64^	PRP superfamily member (SP)
*Ac*_SSH_C_0024	3.5 (0.44)	*A*. *caninum*	BQ666426	1×10^−28^	PRP superfamily member (SP)

Log_2_ ratios are reported for the most highly up-regulated genes as evidenced by microarray. The standard error of the Log_2_ ratio is provided in parentheses. Sequence similarity searches were conducted through dbEST, NCBI, and Swiss-prot using a translated query. (SP) indicates a predicted signal sequence peptide. (PRP) indicates members of the PRP superfamily. PRPs have been termed Ancylostoma secreted proteins (ASPs) in hookworms.

**Table 6:** Most highly down-regulated mRNAs associated with serum stimulation

GeneName	Log_2_ Ratio (SE)	Species	Accession	E-value	Classification
*Ac*_SSH_C_007	−6.0 (0.64)	*A*. *caninum*	AW627014	2×10^−48^	Novel
AW589190	−5.3 (0.49)				(SP)
AW626807	−5.2 (1.50)	*A*. *caninum*	AAC35986	1×10^−61^	Sim. to *Ac*-*asp*-*2* (PRP)
AW589041	−4.8 (0.50)				
AW626929	−4.9 (0.49)				
AW735403	−4.6 (0.32)	*C*. *briggsae*	CAE64405	1×10^−11^	Hypothetical protein cbg09097
*Ac*_SSH_S_109	−4.6 (0.80)	*A*. *caninum*	AW627087	3×10^−67^	Cytochrome P450 Cyp-34A4
*Ac*_SSH_C_015	−4.5 (0.53)	*A*. *caninum*	AW700351	1×10^−44^	*Ac*-*mtp*-*1*
AW782975	−4.4 (1.18)	*C*. *elegans*	AAG24180	5×10^−13^	Hypothetical protein t01g6.10
Contig139	−3.9 (0.29)				
*Ac*_SSH_C_017	−3.9 (0.13)	*N*. *americanus*	AF079521	2×10^−105^	Sim. to *Na*-*asp*-*1* (SP) (PRP)
*Ac*_SSH_C_008	−3.5 (0.32)	*A*. *caninum*	BM077892	5×10^−42^	Alpha-B-Crystallin
*Ac*_SSH_S_038	−3.4 (0.39)	*A*. *caninum*	BG232502	3×10^−63^	Novel
Contig543	−3.4 (0.54)				
AW700711	−3.3 (0.36)				
BQ666059	−3.2 (2.89)				
AW588519	−3.1 (0.21)				
DW718196	−3.1 (1.13)	*H*. *sapiens*	AL160151	1×10^−145^	RP11-168G22 (chromosome 13)
BM077835	−3.0 (0.50)	*C*. *elegans*	CAA92771	5×10^−13^	Heat shock protein 12.6
AW626815	−2.7 (0.27)				
AW700927	−2.7 (0.20)				(SP)
BM077562	−2.6 (0.26)	*C*. *briggsae*	CAE57974	3×10^−27^	Hyp. protein cbg01035
AW782965	−2.5 (0.22)	*C*. *elegans*	CAA98234	4×10^−17^	Hyp. protein c12d8.4
AW181643	−2.5 (0.22)	*D*. *discoideum*	AAC77879	3×10^−15^	ADP/ATP translocase (SP)
*Ac*_SSH_C_115	−2.5 (0.91)	*A*. *ceylanicum*	CB175511	5×10^−79^	Sim. to C-type lectin (Y25C1A) (SP)
BQ667669	−2.4 (0.28)	*C*. *briggsae*	CAE58531	3×10^−42^	Hyp. protein cbg01687
BQ667555	−2.4 (0.19)	*A*. *ceylanicum*	AAN11402	9×10^−40^	PRP superfamily member
BF250627	−2.3 (0.40)	*C*. *elegans*	AAB00621	4×10^−50^	Putative amino acid transporter
AW627137	−2.3 (0.16)				
AW700856	−2.3 (0.18)	*C*. *elegans*	CAA95817	7×10^−17^	Hyp. protein f22d6.11

Log_2_ ratios are reported for the most highly down-regulated genes as evidenced by microarray. The standard error of the Log_2_ ratio is provided in parentheses. Sequence similarity searches were conducted through dbEST, NCBI, and Swiss-prot using a translated query. (SP) indicates a predicted signal sequence peptide. (PRP) indicates members of the PRP superfamily. PRPs have been termed Ancylostoma secreted proteins (ASPs) in hookworms.

**Table 7:** Activation associated proteases

Contig ID	MEROPS	Log Ratio (SE)	Description	Species	GenBank	E
***Metallo-***						
*Ac*_SSH_C_015	MA.M12.A	−4.5 (0.53)*	1) *Ac*-*mtp*-*1*	*A*. *caninum*	AAK62032	2×10^−55^
			2) *Ac*-*mtp*-*1*	*A*. *caninum*	AY036056	1×10^−53^
BQ667149	MG.M24.B	1.4 (0.18)*	1) Hyp. protein cbg15103	*C*. *briggsae*	CAE69085	6×10^−42^
			2) Sim. to XAA-Pro dipeptidase	A. ceylanicum	BM131159	6×10^−69^
BM130304	MA.M13	2.4 (0.17)*	1) *Ac*-*mep*-*2*	*A*. *caninum*	AAG29105	4×10^−54^
			2) Sim to C. elegans F54F11.2	*H*. *contortus*	BE496743	4×10^−53^
*Ac*_SSH_C_0075	MA.M12	2.9 (0.25)*	1) Metalloprotease	*O*. *ostertagi*	CAD28559	3×10^−35^
			2) Metalloprotease	*A*. *caninum*	BQ125213	2×10^−88^
Ac_SSH_C_0109	MA.M12.A	3.3 (0.07)*	1) Metalloprotease-1	*A*. *ceylanicum*	AAN11401	4×10^−64^
			2) Metalloprotease-1	*A*. *ceylanicum*	AY136547	3×10^−59^
***Cysteine***						
BQ125325	CA.C01.A	2.5 (0.23)*	1) Hyp. protein m04g12.2	*C*. *elegans*	CAB03209	5×10^−32^
			2) Sim. to *C*. *elegans cpz*-*2*	*S*. *ratti*	CD421081	3×10^−26^
Ac_SSH_C_0180	CA.C01	2.7 (0.05)*	1) Hyp. protein cbg23351	*C*. *briggsae*	CAE75367	1×10^−137^
			2) Cysteine protease	*A*. *suum*	U51892	3×10^−101^
Ac_SSH_C_0019	CA.C01.A	4.2 (0.34)*	1) *Ac*-*cp*-*1*	*A*. *caninum*	AAC46877	1×10^−100^
			2) Cysteine protease	*H*. *contortus*	Z69345	3×10^−103^
***Aspartyl***						
Ac_SSH_S_0226	AA.A01	2.2 (0.21)*	1) Hyp. protein cbg11305	*C*. *briggsae*	CAE66088	2×10^−36^
			2) Sim. to human cathepsin E	*C. briggsae*	CD421081	3×10^−26^
Ac_SSH_C_0046	AA.A01	2.8 (0.26)*	1) Aspartyl protease-2	*C*. *elegans*	AAO25989	1×10^−123^
			2) Necepsin I	*N*. *americanus*	AJ245458	3×10^−98^
Ac_SSH_S_0165	AA.A01	3.0 (0.53)*	1) Hyp. protein cbg20329	*C*. *briggsae*	CAE72985	2×10^−54^
			2) Aspartyl protease	*P*. *pacificus*	BH836372	5×10^−50^
Ac_SSH_C_0068	AA.A01.A	3.0 (0.07)*	1) Aspartyl protease-1	*C*. *briggsae*	CAE72985	7×10^−60^
			2) Aspartly protease	*A*. *caninum*	BQ666543	1×10^−75^
***Serine***						
BE352528	PA.S01.A	1.8 (0.13)*	1) Mast cell protease 7	*M*. *musculus*	AAH11328	2×10^−15^
			2) Chymotrypsinogen	*X*. *index*	CV512147	2×10^−13^

Log_2_ ratios are reported for activation-associated mRNAs confirmed by microarray. The standard error for each log_2_ ratio is provided in parentheses. Protease classifications were determined from the MEROPS database and are reported here in the [Family.Clan.Subfamily] format. 1) Results from tblastn (GenBank NR). 2) Results of tblastx (nematode dbEST).

### Time course of activation-associated mRNAs

Selected mRNAs were examined at different time points throughout the course of serum stimulation (Table 8). Four mRNA's were up-regulated and 4 were down-regulated within 1 hour of stimulation, then a further 80 responded to stimulation after 6–13 hours.The mRNAs that were rapidly up- or down-regulated following activation included the heat shock protein gene, *hsp* 12.6, the cysteine protease *Ac*-*cp1*, a metalloprotease and an mRNA similar to the *Onchocerca* related antigen (*ora*-*1*) from *C*. *elegans*. Several PRPs, as well as the metalloprotease *Ac*-*mtp*-*1* and a neuropeptide-like protein exhibited obvious differential transcription after at most 6 h. Interestingly, *Ac*_SSH_0042_A, one of the most highly up-regulated activation-associated PRPs, did not appear to increase in transcription until after 13 h of incubation.

**Table 8:** Transcription of activation-associated mRNAs

Sequence		Log_2_ ratio (SE)			
		1 hour	6 hours	13 hours	24 hours
*Ac*_SSH_C_0042_A	PRP superfamily member	0.6 (0.05)	0.3 (0.03)	4.8 (0.43)	8.9 (2.49)
*Ac*_SSH_C_0008_A	PRP superfamily member	−0.2 (0.02)	2.4 (0.18)	5.9 (0.65)	6.7 (0.43)
*Ac*_SSH_C_0109	Metalloprotease	1.5 (0.13)	2.0 (0.17)	2.1 (0.22)	6.2 (0.96)
*Ac*_SSH_C_0019	*Ac*-*cp1* (cysteine protease)	1.9 (0.17)	6.1 (0.60)	6.2 (0.60)	5.9 (1.39)
*Ac*_SSH_C_0069	Weak sim. To boophilin	3.4 (0.47)	5.7 (0.83)	5.8 (0.79)	5.9 (0.61)
*Ac*_SSH_C_0099	Novel mRNA	2.1 (0.21)	3.4 (0.35)	4.8 (0.42)	5.0 (0.31)
*Ac*_SSH_C_0017	PRP superfamily member	−0.5 (0.03)	4.2 (0.31)	5.5 (0.49)	5.0 (0.35)
*Ac*_SSH_C_0056	*O*. *volvulus related antigen*	−1.4 (0.14)	1.7 (0.18)	2.0 (0.22)	3.5 (0.88)
*Ac*_SSH_C_0032	Novel mRNA	−0.2 (0.01)	0.9 (0.05)	1.2 (0.08)	2.8 (0.19)
*Ac*_SSH_C_017	PRP superfamily member	−0.2 (0.01)	−2.4 (0.23)	−3.2 (0.25)	−3.4 (0.19)
*Ac*_SSH_C_015	*Ac*-*mtp*-1 (metalloprotease)	0.0 (0.00)	−2.5 (0.18)	−2.5 (0.12)	−3.1 (0.19)
*Ac*_SSH_C_007	Novel mRNA	−1.7 (0.13)	−4.1 (0.35)	−2.6 (0.18)	−3.0 (0.24)
*Ac*_SSH_C_008	Alpha-beta crystaline	−1.7 (0.08)	−2.9 (0.15)	−2.2 (0.11)	−2.3 (0.20)
*hsp*-*12.6*	Heat shock protein 12.6	−2.8 (0.32)	−3.9 (0.38)	−3.3 (0.29)	−2.1 (0.43)
*Ac*_SSH_S_105	Weak sim. to a C-type lectin	−0.3 (0.03)	−1.0 (0.10)	−0.8 (0.08)	−1.3 (0.15)
*Ac*_SSH_C_018	Neuropeptide-like protein	0.3 (0.02)	−1.3 (0.08)	−0.6 (0.05)	−1.2 (0.14)

The expression of selected activation-associated mRNAs and *hsp*-*12*.*6* were examined for each time point. All log_2_ ratios are relative to the non-activated controls and standard errors of the mean are provided in parentheses. The number of individual observations for the 24 hour and non-activated controls was at least 24. The statistics for all other time points are based on at least 6 separate measurements.

### Gene ontology analysis

**Figure 4:**
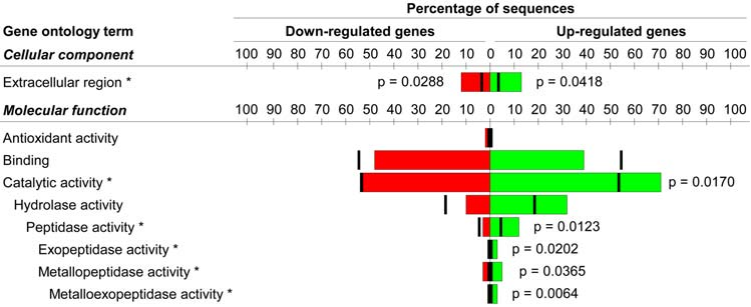
Gene ontology (GO) annotations to activation-associated genes. GO annotations for up- and down-regulated activation associated mRNAs were tabulated and summarized using high-level terms. GO terms were also tested for statistically significant over-representation (P<0.05). Over-represented terms are provided here in the context of their parent terms. Red and green bars indicate the observed distribution of a particular GO term, whereas black ticks denote the expected distribution based on observed frequencies in the entire known (publicly available) EST data for *A. caninum*. All P-values reported are not adjusted for multiple testing correction but are only provided for terms which are below the “false discovery” rate of 0.5.

All activation-associated mRNAs were annotated with GO terms based on sequence similarity using the Blast2GO platform, and 3-level summaries were prepared for each aspect of GO, molecular function, biological process and cellular component (Figure 4). It is important to note that these classifications provide an estimation only of gene function, because the sequence data used are mRNAs, and often only partial sequences. Nonetheless, we identified a number of gene families that were highly upregulated in activated L3s. The GO category for catalytic activity was significantly over-represented in the mRNAs which were up-regulated upon serum stimulation. Inspection of higher-level terms in the GO tree showed that this significance was likely accounted for by the many proteases and other hydrolytic enzymes which were up-regulated during serum stimulation (Table 7). Based on the frequency of proteases encoded by the current gene/cDNA entries (n = 48) for *A. caninum* in the NCBI databases, only three such molecules would be expected among the 66 annotated, up-regulated mRNAs. Instead, a total of seven were observed. Additionally, three of the 13 genes predicted to encode metallopeptidases were up-regulated in activated L3. The importance of proteases in activation seems evident from Figure 4, which shows that a majority of the up-regulated genes encode ‘protein catabolism’ functions, a sub-category of the ‘biological process’ GO category.

The GO data were also analysed in the context of mRNA expression data [29]. This analysis focused on the degree to which mRNA expression within specified GO categories was greater than the global array average (Table 9). The absolute log_2_ ratios of genes associated with defence and the response to external stimuli were 2.4 to 2.9 times greater than the average. The log_2_ ratio of genes encoding proteins with a predicted extracellular localization was greater than three times the average, with this trend being reflected primarily by the PRPs and proteases. Interestingly, mRNAs associated with carbohydrate binding were more highly represented than average (EQ = 2.7). The largest group of annotated genes was associated with catalytic activity. Collectively, these genes had log_2_ ratios, which were 1.3 times greater than the average. However, hydrolases and lyases were largely responsible for the trend. Hydrolases (n = 93) and lyases (n = 14) were generally up-regulated 1.5 to 1.7 times more than the average, which was considerable given the size of those GO categories.

**Table 9:** Annotation-specific expression quotients

Term	n	N	EQ (SE)
Biological process	372	747	
Cellular process	338	684	1.05 (0.04)*
Cell adhesion	3	7	1.87 (0.57)
Physiological process	344	697	1.08 (0.05)*
Organismal physiological process	11	27	1.75 (0.38)*
Response to stimulus	26	41	1.06 (0.14)
Defense response	4	6	2.38 (0.60)*
Physiological response to stimlus	10	14	1.48 (0.30)
Response to external stimulus	3	7	2.83 (0.81)*
Cellular component	302	621	
Extracellular region	14	26	3.33 (0.51)*
Extracellular region part	4	6	1.63 (0.59)
Extracellular space	3	5	2.12 (0.76)
Molecular function	485	927	
Binding	270	504	0.93 (0.03)
Carbohydrate binding	4	7	2.73 (0.72)*
Catalytic activity	275	503	1.29 (0.05)*
Hydrolase activity	93	176	1.69 (0.14)*
Lyase activity	14	23	1.54 (0.22)*
Enzyme regulator activity	8	16	0.79 (0.19)
Enzyme inhibitor activity	2	10	1.49 (0.82)
Motor activity	3	7	1.78 (0.67)
Microtubule motor activity	2	3	1.50 (0.75)
Structural molecule activity	23	96	0.68 (0.09)
Structural constituent of cytoskeleton	3	5	1.61 (0.48)
Transporter activity	48	96	0.98 (0.10)
Organic acid transporter activity	3	5	1.87 (0.79)
Protein transporter activity	2	5	2.07 (1.52)

Expression quotients were calculated for each 3-level gene ontology term mapped to activation-associated genes. The quotients are calculated as the mean of the absolute log_2_ ratio of a category divided by the global log_2_ ratio of the chip. A single asterisk indicates that the 95% confidence interval for the quotient did not include 1. Differences observed in n (number of genes with array data) and N (number of annotated genes in the suppression subtractive hybridization and public EST databases) are due in combination to probe failure as well as an inability to produce unique probes for some genes.

**Figure 5:**
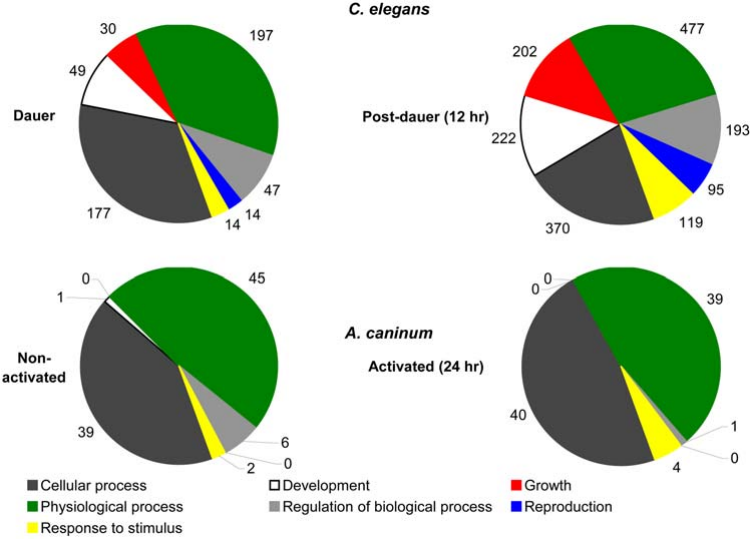
Comparison of gene ontology (GO) terms associated with analogous processes. Two-level GOs with respect to biological process for activated *A. caninum* and *C. elegans* 24 h post-dauer exit.

The GO classifications can be useful for functional comparisons among species. A comparison was made between activated L3 in *A. caninum* and *C*. *elegans* larvae exiting from dauer. The 3-level charts (Figure 5) display the distribution of GO terms specific to the category of biological process for the 30–36% of hookworm ESTs where a GO function could be assigned. While the “post-stimulation” transcriptome of both organisms was dominated by genes associated with cellular and physiological processes, it was evident that dauer exit in *C*. *elegans* was associated with a substantial increase in the proportion of genes involved in growth, development and reproduction. By comparison, serum stimulation in *A*. *caninum* did not result in an increased representation of these mRNAs. Interestingly, even the pool of annotated sequences from *C. elegans* dauer larvae included (15%) of mRNAs associated with development. This was not the case for the ensheathed, non-activated L3 of *A*. *caninum*.

## Discussion

For many parasitic nematodes, developmental arrest at the L3 stage is critical for their survival in the environment. In the hookworm model, the exit from developmental arrest is associated with the invasion of the host, assessment of host suitability (larval hypobiosis *vs.* development to adulthood) and evasion of the host immune response. Therefore, it is plausible that genes associated with this exit facilitate these processes. Using a SSH-based EST approach, we explored transcription in the L3 stage of *A. caninum* during the transition from a free-living to a parasitic larva, by simulating the earliest stage of parasitism by hookworms in the mammalian (canine) host *via* serum stimulation. The ESTs produced using this approach were then incorporated into a customised oligonucleotide microarray together with a set of known *A*. *caninum* sequences, in order to carry out a large-scale analysis of transcripts during this transition to parasitism.

### Pathogenesis-related proteins

High-level GO summaries demonstrated that a large proportion (12%) of differentially expressed mRNAs appear to be involved in extracellular localization, of which the majority (27) encode a group of proteins belonging to the PRP superfamily. The greater than average transcription of genes encoding PRPs highlights their importance in the transition of *A. caninum* from a free-living to a parasitic larva. Although the functions of these molecules are largely unknown, the identification of eight *A*. *caninum* PRP superfamily members from the excretory/secretory products of larvae [13],[30] and adults [31]–[33] suggests involvement in host-parasite interactions. Consistent with this hypothesis is the observation that the *N*. *americanus* orthologue of *Ac*-ASP-2, a major vaccine antigen from *N*. *americanus* [34], possesses a crystal structure similar to a chemokine, suggesting that it may serve as an extracellular ligand for an unknown host receptor involved in inflammation [35]. Furthermore, *Ac*-NIF (neutrophil inhibitory factor) [33] and *Ac*-HPI (platelet inhibitor) [32], the only two *A*. *caninum* PRPs for which *in vivo* functions have been proposed, both exhibit *in vitro* activities in the mediation of the inflammatory response. All eight of the *A*. *caninum* PRPs characterized to date are secreted proteins. Similarly, the presence of predicted signal peptides in most of the activation-associated PRPs suggests that they too are secreted.

### Activation-associated proteases

Although most of the mRNAs encoding extracellular proteins were PRPs, others encoded proteases. The importance of the activation-associated proteases was evident in the high-level GO summary of differentially expressed mRNAs (Table 9). In broad terms, catalytic activity was over-represented in mRNAs from activated L3, with proteases largely reflecting this trend. The activation-associated proteases represented four of the major catalytic families, namely the metallo-, aspartic, cysteine and serine proteases. These proteases may serve roles in host tissue degradation, digestion and/or development. For example, the activation associated mRNA *Ac*_SSH_C_0180 is a likely homologue of *Parelaphostrongylus tenuis cpl*-*1*, which encodes a cysteine protease implicated in the digestion of host tissue during the escape of the L3 from the intermediate snail host [36]. While many mRNAs encoding proteases were upregulated upon activation, a few such as those encoding the astacin-like metalloprotease, *Ac-*MTP-1, were down-regulated. MTP-1 is thought to play a critical role in skin penetration *in vivo* [26], thus supporting its candidacy as a vaccine antigen [37]. A similar scenario exists in larval schistosomes, where the major protease involved in tissue penetration is pre-synthesized and its mRNA is down-regulated before the cercarial stage infects the mammalian host (reviewed in [38]).

Proteases also serve roles in nematode development. For example, *O*. *volvulus* cathepsin Z (*Ov*-*cpz*-*1*) is expressed in the cuticle of *O*. *volvulus* and is essential for the moult of the L3 to L4 stage [39]. Evidence suggests that the *cpz*-*1* orthologue in *C*. *elegans* is also necessary for normal moulting and development [40]. The activation-associated *A*. *caninum* mRNA, BQ125325, is cathepsin Z-like and could therefore fulfil a similar role in the moult of hookworm L3 to L4. In addition to moulting, the beginning of the exit from dauer in *C*. *elegans* involves a major neurological restructuring [41]. The aspartyl protease *Ce*-ASP-2 plays an important role in neurodegeneration in this species [42], and the association of a likely hookworm orthologue (*Ac*_SSH_C_0068) with serum stimulation may also indicate a role in neurological development.

Lastly, other activation-associated proteases may be involved in the digestion of host proteins for nourishment. *Ac*_SSH_C_0046 is 60% identical to *H*. *contortus* pepsinogen (CAA96571) and *necepsin I* (also referred to as *Na*-APR-2) from *N*. *americanus* (CAC00542.1) [43]. The pepsinogen of *H*. *contortus* is expressed in the gut of the adult stage, and mRNAs have been detected in the L4 and adult stages but not in the L3 [44]. Furthermore, its ability to degrade haemoglobin indicates that it could be involved in feeding [44]. The *N. americanus* aspartyl proteases *Na*-APR-2, *Na*-APR-1 and the *A*. *caninum* orthologue, *Ac*-APR-1, are all expressed in the gut of the adult stage where they digest haemoglobin [45].

### Comparisons with *C*. *elegans*

Mitreva et al. [8] observed that nearly 80% of the *A. caninum* clusters that were publicly available (before our study here) shared some degree of significant sequence similarity with *C*. *elegans* sequences. Moser et al. [7] compared the “serum-stimulated expression data” from many of these clusters to the 1,984 mRNAs associated with dauer exit in *C*. *elegans*. This was done on a gene-by-gene basis, and it was observed that cytochrome P450, two neuropeptides, phospholipase and alcohol dehydrogenase were enriched in the dauer form of *C. elegans* and in non-activated L3 of *A. caninum* [7]. Conversely, these authors identified mRNAs representing cytochrome *c* oxidases, an arginine kinase, a heat shock protein, a glycerol hydrolase and glyceraldehyde 3-phosphate dehydrogenase, which were up-regulated in both nematode species following stimulation. Our findings are in accordance with these reports. Neither our study nor that of Moser et al. [7] identified major similarities between the “activated” states of *C*. *elegans* and *A*. *caninum*. This was attributed to the fact that many of the *C*. *elegans* genes which were up-regulated were under-represented in the *A*. *caninum* dataset [7]. However, even after enriching for mRNAs that are differentially expressed between free-living and activated L3, the lack of similarity between recovered dauers and activated hookworm L3 persisted.

*In lieu* of a gene-by-gene approach, we used species-independent GOs to assess the similarity of the relevant *A*. *caninum* and *C*. *elegans* transcriptomes. This analysis made use of the microarray data generated by [46]. This comparison demonstrated that GO annotations specific to growth, development and reproduction were highly represented in the recovered dauers of *C*. *elegans* (Figure 5). This was not the case in *A*. *caninum* and is consistent with the observation that serum-stimulation does not invoke moulting of the L3 stage [47]. Conversely, mRNAs from *C*. *elegans* dauers or larvae recovered 12 h after beginning of the exit from dauer did not exhibit the significant over-representation of extracellular products as was observed for *A*. *caninum*. This finding supports the hypothesis that many of the highly up-regulated mRNAs encoding putatively secreted products are involved in parasitism.

Another major difference between “activation” in *C*. *elegans* and *A*. *caninum* is the down-regulation of several mRNAs encoding genes involved in G-protein coupled signal transduction during dauer exit in *C*. *elegans*. Such a down-regulation was not observed for *A*. *caninum* in the present study or that of [7]. As opposed to the PRPs of activated *A*. *caninum* L3, the predominant transcripts in *C*. *elegans* 12 h after beginning the exit from dauer included a plethora of collagens, many of which were up-regulated ≥32-fold [46]. Even after enrichment for activation-associated mRNAs, only three potential collagens were identified from *A*. *caninum* and only one of these (cuticulin) was significantly up-regulated. Based on this information, the activation of *A. caninum* larvae and the exit from dauer involve considerably different mRNAs. However, the mechanisms by which these mRNAs are regulated may be similar. *Ce*-*hsp*-*12.6* is a well-known direct target of the fork head transcription factor (designated DAF-16) in *C*. *elegans* [48]. Transcripts for this gene are down regulated during dauer exit. Interestingly, it was observed that the *A*. *caninum* orthologue of *hsp*-*12.6* (contig 313 from the publicly available ESTs) was also significantly down-regulated during serum stimulation. Assuming that the transcription of this gene is also under the direct regulation of a DAF-16 homologue, the earliest transcriptional events in the transition of *A. caninum* L3 to parasitism may also be regulated by DAF-16. Real-time PCR conducted on several activation-associated mRNAs at various time points throughout the serum-activation process showed that the levels of many of these mRNAs changed rapidly, as half of those assessed achieved log_2_ ratios of noticeably more than zero in less than 1 h. The *hsp-*12.6 transcript was represented in this group of “early responder” molecules. Other mRNAs with similar expression profiles may also be directly regulated by DAF-16. Interestingly, one of the most highly up-regulated PRP mRNAs did not increase substantially in transcription until 13 h after serum-stimulation, which suggests that it may be under the indirect control of DAF-16.

### Genes of unknown function

The SSH enrichment of activation-associated mRNAs identified 17 sequences which were up-regulated ≥4-fold and appeared to be unique to parasitic nematodes (Table S1). For example, the EST *Ac*_SSH_C_0056 was similar in sequence to an uncharacterised gene, *ora*-*1*, from *C*. *elegans* which is related to an *O*. *volvulus* antigen (Ov39) and is thought to play a role in the ocular pathogenesis caused by this parasite [49]. The mRNA representing *Ac*_SSH_S_0199 was up-regulated nearly 9-fold with the EST showing sequence similarity to the genes *Hc*-*nim*-1 and *Hc*-*nim*-*2* from *H*. *contortus*. The mRNAs encoding these genes are abundant in adult *H. contortus* and represent almost 10% of total mRNA [50]. *Hc*-NIM-1 is expressed in the hypodermis of the pharyngeal region of the adult worm [50]. Lastly, SSH contigs 0099 and 0032 were among the most highly up-regulated mRNAs in activated L3 of *A. caninum*, with log ratios of ∼4.7 and 5.9, respectively. Both were predicted to possess a signal peptide and appeared to be specific to *A*. *caninum*. Their apparent novelty and stage specificity suggest that they are parasite-specific molecules which might be involved in interactions with host tissues. Functional characterization of these and other novel activation-associated mRNAs may provide insights into the roles that such molecules play in the transition to parasitism. Furthermore, this information may warrant investigating their potential as targets for novel therapeutics.

### Conclusions

Having identified a suite of mRNAs associated with serum stimulation, future efforts should be focused on gaining an understanding of the biological function/s of selected members of these parasitism-associated genes. Of particular interest is the large group of PRPs that are up-regulated upon serum stimulation. In combination with their considerable stage specificity and diversity, many of these PRPs may have evolved to perform several coordinated yet distinct functions involved in the parasitic process. Given that PRP-like proteins occur in a wide range of taxa, delineating their function could potentially provide a deeper insight into their roles in parasitism as well as their broader biological significance. It is also of interest that the two most efficacious hookworm vaccine antigens, ASP-2 and APR-1, are members of the two most represented families/groups of proteins associated with this transition to parasitism, PRPs and proteases. We believe that this bodes well for the pursuit of these new molecules identified by SSH as targets for novel vaccines and drugs.

The near absence of mRNAs associated with reproduction, growth and development among activated hookworm L3 probably reflects their ability to further arrest in tissues of non-permissive hosts or in the external environment when conditions for transmission are unfavourable. Although this should not invalidate *C*. *elegans* dauer exit as a model for hookworm activation, it highlights the limitations of this free-living nematode as a model organism for the transition of nematode larvae from a free-living to a parasitic state.

## Supporting Information

Figure S1 Magnitude (M) versus Amplitude (A) plot of dye-swap self-hybridisations. RNA from serum-activated third-stage larvae (L3) of *A. caninum* was labelled with Cy3 or Cy5 for “self” hybridisation. The resultant log ratios were plotted against the log2 of the mean signal intensity, thus providing a visual means of inspecting potential artefacts for a range of signal intensities. A self-hybridisation was also performed for RNA extracted from non-activated L3 as a control.
(0.08 MB PPT)

Table S1 Up-regulated activation associated mRNAs apparently unique to nematodes
(0.03 MB DOC)

Table S2 Primer sequences used for real-time PCR analysis
(0.03 MB DOC)
