# Supplementary figures and images for: Transcriptional Changes in the Hookworm, Ancylostoma caninum, during the Transition from a Free-Living to a Parasitic Larva
Source: PLoS Negl Trop Dis. 2008 Jan 9;2(1):e130. doi: 10.1371/journal.pntd.0000130 (PMC2217673; doi:10.1371/journal.pntd.0000130)

## Slide 1
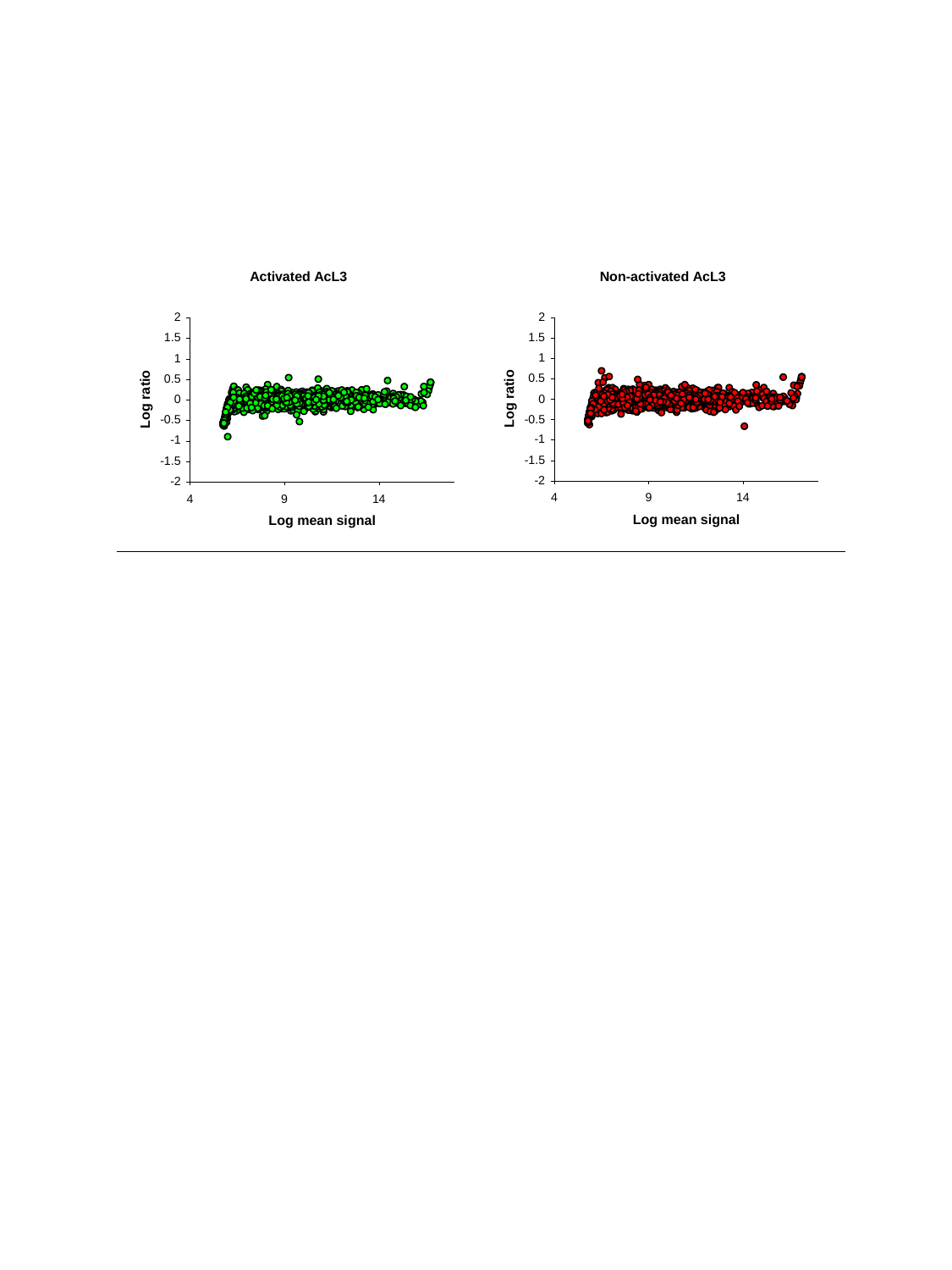

Supplement: Figure S1 — Magnitude (M) versus Amplitude (A) plot of dye-swap self-hybridisations. RNA from serum-activated third-stage larvae (L3) of A. caninum was labelled with Cy3 or Cy5 for “self” hybridisation. The resultant log ratios were plotted against the log2 of the mean signal intensity, thus providing a visual means of inspecting potential artefacts for a range of signal intensities. A self-hybridisation was also performed for RNA extracted from non-activated L3 as a control. (0.08 MB PPT) [file pntd.0000130.s001.ppt]
