# Supplementary material for: Transcriptional Changes in the Hookworm, Ancylostoma caninum, during the Transition from a Free-Living to a Parasitic Larva
Source: PLoS Negl Trop Dis. 2008 Jan 9;2(1):e130. doi: 10.1371/journal.pntd.0000130 (PMC2217673; doi:10.1371/journal.pntd.0000130)
Supplement: Table S1 — Up-regulated activation associated mRNAs apparently unique to nematodes (0.03 MB DOC) [file pntd.0000130.s002.doc]

**Table S1** Up-regulated activation associated mRNAs apparently unique to nematodes

Several activation-association mRNAs were identified by SSH which appeared to be nematode-specific. Those that are up-regulated following serum stimulation are presented here.
