# Supplementary material for: Transcriptional Changes in the Hookworm, Ancylostoma caninum, during the Transition from a Free-Living to a Parasitic Larva
Source: PLoS Negl Trop Dis. 2008 Jan 9;2(1):e130. doi: 10.1371/journal.pntd.0000130 (PMC2217673; doi:10.1371/journal.pntd.0000130)
Supplement: Table S2 — Primer sequences used for real-time PCR analysis (0.03 MB DOC) [file pntd.0000130.s003.doc]

**Table S2** Primer sequences used for real-time PCR analysis

Two different primer sets were synthesized for SSH Contig 0109 for an indication of the robustness of real-time PCR results.
